# Management of a Traumatic Penetrating Cardiac Injury in a Low-Resource Center Without a Cardiothoracic Surgery Department

**DOI:** 10.7759/cureus.56539

**Published:** 2024-03-20

**Authors:** Jaime T Lee Young

**Affiliations:** 1 Surgery, Port of Spain General Hospital, Port of Spain, TTO

**Keywords:** low resource setting, stab wounds, caribbean trauma, cardio thoracic surgery, penetrating cardiac injury, emergent general surgery

## Abstract

Traumatic penetrating cardiac injury is a rare pathology with a high mortality rate, more commonly occurring in a military setting or during violent assaults in a civilian environment. Given the anatomy, these injuries are often managed by cardiothoracic surgeons. However, in an institute that lacks these specialists, the responsibility for managing this condition falls on the shoulders of the general surgeon on call. We herein report a case where a penetrating cardiac injury was managed successfully by general surgeons in the absence of cardiothoracic surgeons. This case serves two educational purposes. The first is that Caribbean hospitals possess the potential to match a developed country's medical standard if additional resources can be obtained from their respective governing bodies. The second is that a general surgeon’s role is not yet finished in the modern era of sub-specialization, especially in a setting that lacks dedicated specialists.

## Introduction

Penetrating cardiac injury occurs when there is trauma that pierces the heart, usually from a blade or gunshot. The heart is a well-protected organ surrounded by the rib cage. It serves one of the most important functions of the human body, acting as a pump for the circulatory system. Injury to cardiac structures is, therefore, a rare and potentially life-threatening event, with an incidence of approximately 1 in 100,000 persons [1]. Close to 90% of patients with penetrating cardiac wounds do not actually reach the hospital [2], with the most common causes of death being exsanguination, cardiac tamponade, or coronary artery damage [3].

Given that not all institutes may have a cardiothoracic surgeon on call, it is imperative that the general surgeon at these facilities be familiar with the approach and management of cardiac injuries. This is especially true in the Caribbean, where there is a growing increase in violent crime as well as a relative shortage of specialists. The objective of this case report is to highlight that a positive result can be obtained from the timely operation of a cardiac injury, even in a low-resource setting, without specialist intervention.

## Case presentation

**Figure 1:**
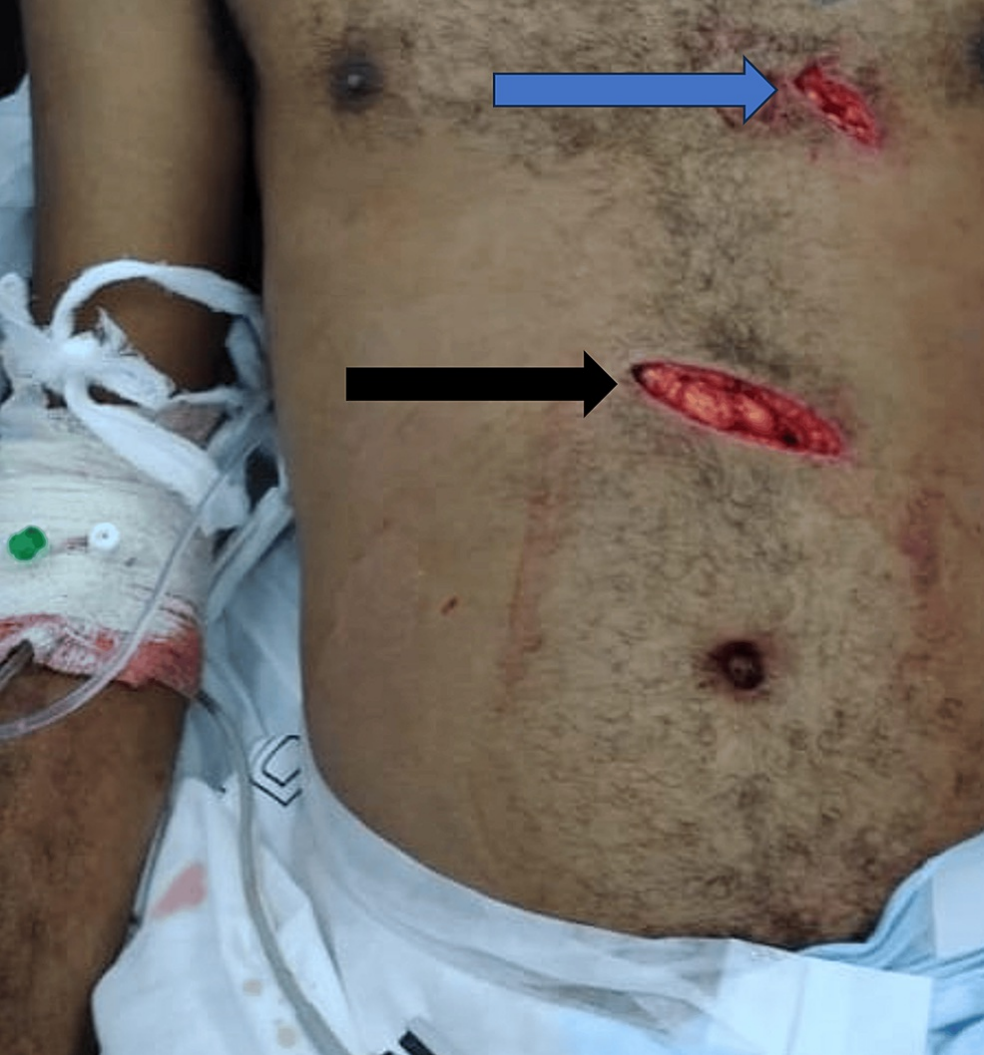
The patient's wounds on presentation to the emergency department. The blue arrow indicates the 3 cm stab wound to the thorax. The black arrow indicates the 6 cm stab wound to the epigastric region of the abdomen.

A 40-year-old male with no known co-morbidities and a shellfish allergy presented to our emergency department less than 15 minutes after an assault with a machete. The blood pressure on arrival was 101/71 mmHg, with a heart rate of 59 beats per minute, a SpO_2_ of 98%, and a respiratory rate of 20 breaths per minute. The patient reported being stabbed in the abdomen and chest during an attempted robbery. On general observation, the patient was of appropriate body habitus, diaphoretic, and in cardiopulmonary distress. A single 3-cm-long elliptical wound was found on the left sternal border at the level of T4 with associated effervescence. A larger, 6-cm-long elliptical wound with a fascial defect was found in the epigastric region, just inferior to the xiphoid process. These wounds are shown in Figure 1.

**Figure 2:**
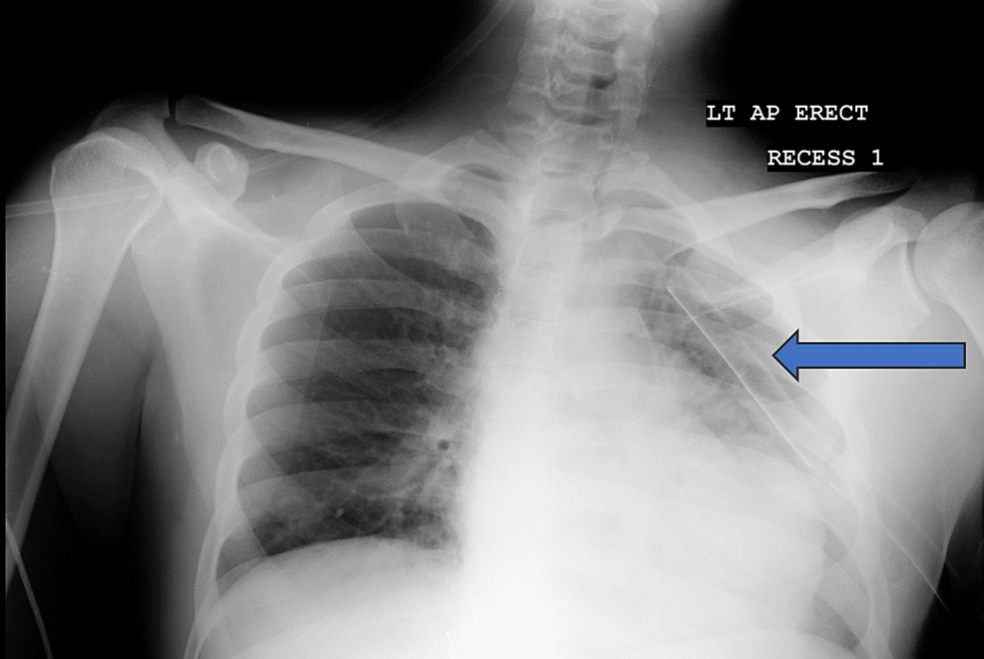
Pre-operative chest X-ray done in the emergency department after insertion of the intercostal chest tube into the left pleural space. The blue arrow indicates the intercostal chest tube.

Following advanced trauma life support (ATLS) guidelines, a systematic team approach was taken, with physical examination and management performed concomitantly. The cardiovascular examination of the patient was normal, and a respiratory examination revealed decreased breath sounds in the lower and middle zones of the left lung that were associated with dullness on percussion. The abdominal examination was significant for generalized peritonitis, active bowel sounds, and a negative rectal examination. Supplemental oxygen was provided via a facemask at 10 liters per minute, and urinary catheterization was performed with the retrieval of 50 ml of straw-colored urine. Bilateral 18-gauge intravenous catheters were placed in the antecubital fossae with the initiation of isotonic crystalloids. A 34 French intercostal tube was inserted in the left fifth intercostal space, just anterior to the midaxillary line, with 300 ml of blood returned. An extended focused assessment with sonography for trauma (eFast) found a sliver of fluid in Morrison’s pouch, a left-sided hemopneumothorax, and no pericardial effusion. The patient’s pre-operative complete blood count and renal function tests are listed in Table 1. A group and cross-match sample were also sent to the laboratory. The patient’s chest X-ray on admission is shown in Figure 2.

**Table 1:** Pre-operative complete blood count and renal function test done in the emergency department.

Laboratory investigation	Result	Normal range
White blood cell count	10.3 × 10^9^ /L	4.0 × 10^9^ to 10.0 × 10^9^/L
Platelets	235 x 10^9^ /L	150 × 10^9^ to 410 x 10^9^ /L
Hemoglobin	15.5 g/dL	13.0–17.0 g/dL
Blood urea nitrogen	9.0 mg/dL	6.0–23.0 mg/dL
Creatinine	0.8 mg/dL	0.7–23.0 mg/dL
Sodium	143 mmol/L	135.0–145.0 mmol/L
Potassium	3.6 mmol/L	3.5–5.1 mmol/L

**Figure 3:**
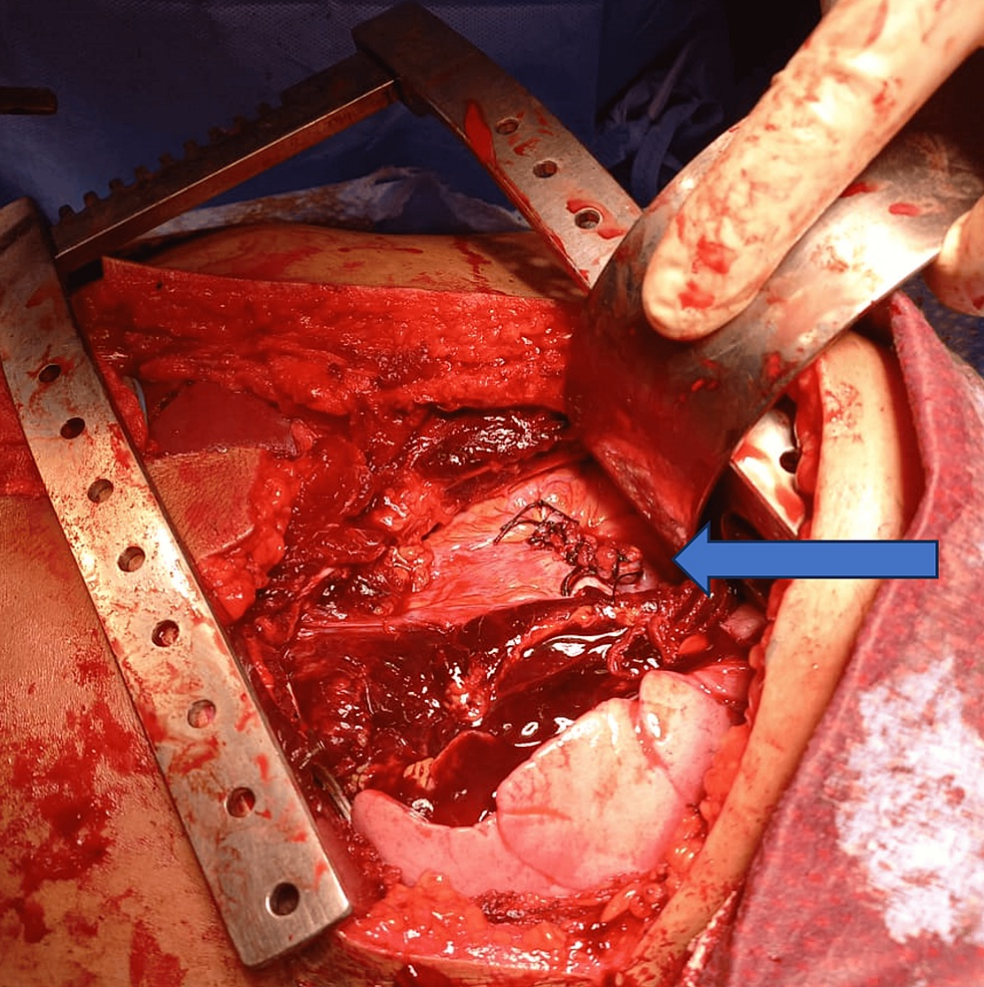
Successful cardiorraphy of the 3 cm right atrial laceration. The blue arrow indicates the suture line of the repaired right atrial laceration.

The patient was taken to the operating theater for an exploratory laparotomy and repair of the fascial defect. A midline incision was made with 200 ml of blood found in the peritoneum with a 4 cm laceration to the left lobe of the liver. After hepatic packing and direct manual compression, hemostasis was obtained. During the inspection of the intestines for injuries, the patient’s vitals rapidly deteriorated. He was found to have a systolic blood pressure of 59 mmHg, no recordable diastolic pressure, and an absent pulse. An intraoperative transthoracic echocardiogram was performed by anesthesiology, revealing a pericardial effusion and decreased contractility of the ventricles. A transdiaphragmatic pericardial window was performed with the return of approximately 400 ml of pulsatile bleeding that subsequently slowed and was associated with an improvement in the patient’s blood pressure. A left anterolateral thoracotomy was immediately performed, and the pericardial sac was opened anterior to the phrenic nerve. A 3 cm laceration was found in the right atrium. Digital occlusion of the laceration was performed while allis clamps were applied to oppose the wound edges. It was repaired with 2-0 silk in a running horizontal mattress suturing technique. Figure 3 shows the completed cardiorraphy.

A 15-French drain was placed in the pericardial sac and passed through the anterior abdominal wall via the transdiaphragmatic pericardial window. The pericardial sac was subsequently repaired with 2-0 vicryl. Intercostal muscles were also reapproximated with 2-0 vicryl in layers. The peritoneum was irrigated, with the remainder of the abdominal contents having sustained no injuries. Another 15 French drains were left intra-abdominally, the anterior abdominal wall was closed in layers, and a nasogastric tube was inserted. An arterial blood gas performed during the peri-arrest revealed uncompensated metabolic acidosis, and the results are shown in Table 2.

**Table 2:** Results of the arterial blood gas done during the intra-operative peri-arrest period revealing uncompensated metabolic acidosis.

Laboratory investigation	Result	Normal range
pH	7.125	7.35–7.45
pCO_2_	43.6 mmHg	32.0–48.0 mmHg
SO_2_	100%	94.0–98.0%
Base excess	−14.37 mEq/L	−2.0 to 2.0 mEq/L
HC0_3_	14.0 mEq/L	22.0–28.0 mEq/L

Subsequently, the patient was transported to the intensive care unit (ICU), intubated, and sedated on midazolam 50 mg/50 ml and ketamine 500 mg/50 ml. He was also started on ceftriaxone 1 g IV BD and Nexium 40 mg IV OD. His blood pressure on arrival was 149/90 mmHg, his heart rate was 87 beats per minute, and his respiratory rate was 20. An arterial blood gas done later that day revealed improvement in metabolic acidosis.

On postoperative day 1, he remained hemodynamically stable without pressure support. An electrocardiogram revealed ST segment elevations in leads 1, 2, aVL, V2, V4, V5, and V6; a diagnosis of traumatic pericarditis was made. Colchicine 0.5 mg PO OD was planned to be started; however, the hospital pharmacy reported that no colchicine was available in the institute. He remained intubated and sedated on postoperative day 2 in the ICU. Enoxaparin sodium 40 mg SC OD and aspirin 81 mg PO OD were both started.

**Figure 4:**
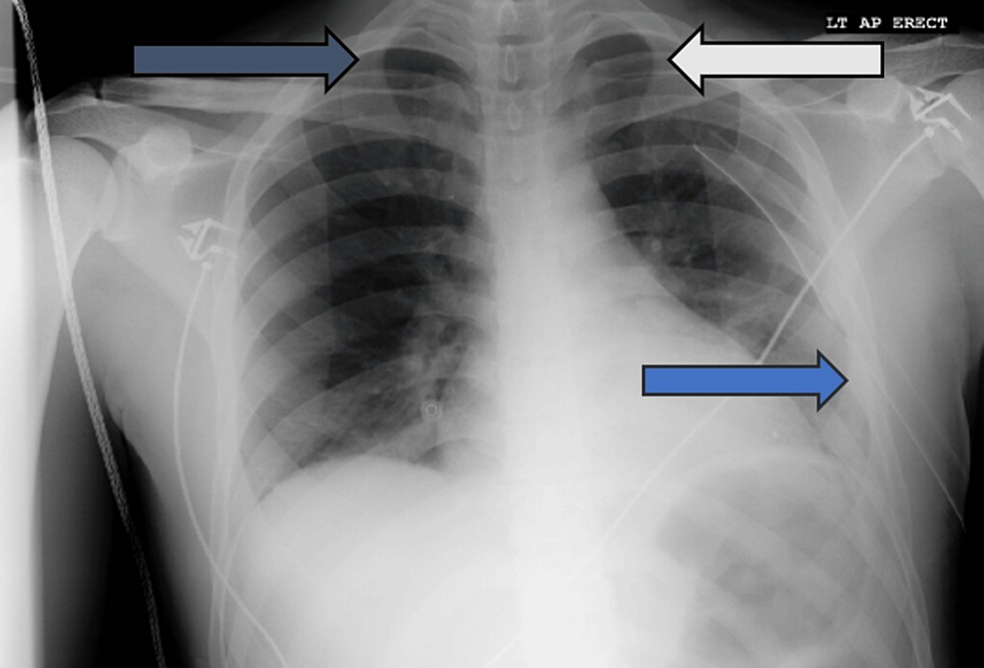
Chest X-ray done on post-operative day 8. The blue arrow indicates the chest tube inserted on presentation to the emergency department is still in the correct position. The white arrow indicates a clear left lung field with no associated pneumothorax or hemothorax. The gray arrow indicates a clear right lung field with no associated pneumothorax or hemothorax.

On day 4 of the patient’s ICU stay, he was extubated without incident. No neurological deficits were found; he was oriented once fully awake and able to follow instructions. An echocardiogram revealed normal systolic function of the left and right ventricles, normal regional and global wall motion, trivial tricuspid regurgitation, normal mitral, aortic, and pulmonary valves, as well as nil pericardial effusion. His nasogastric tube was clamped, and he was allowed to have sips of clear fluids, which he tolerated with no nausea or vomiting. The abdominal drain was removed without complications after having minimal drainage. On postoperative day 8, a chest X-ray was performed that showed no pneumothorax or hemothorax, and the chest tube was removed. The chest X-ray is shown in Figure 4. He was discharged on postoperative day 10 on ASA 81 mg PO OD and omeprazole 20 mg PO OD and was discharged with instructions to return for outpatient follow-up at the surgical clinic.

## Discussion

Cardiac injuries are classically associated with penetrating wounds to the cardiac box. This box is an anatomic region bounded superiorly by the clavicles, laterally by the midclavicular lines, and inferiorly by the xiphoid [4]. Stab wounds within the boundaries of the "box" have been found to have a higher risk of cardiac injury [5]. The etiology of the injury is of significant importance to the outcome of the patient, with gunshot and stab wounds having a survival rate of 9.7% and 32.6%, respectively [1].

Gunshot injuries are associated with a significant mortality rate due to the greater kinetic energy they possess compared to stab wounds. The tumbling and fragmentation of the bullet as it travels through tissue result in a permanent cavity that results in injury to structures along the trajectory of the bullet. A temporary cavity, also known as cavitation, also occurs due to an expanding bubble of vapor from the bullet that can result in injury to structures not directly along the bullet’s trajectory. The phenomenon of cavitation is directly proportional to the speed of the bullet; it is therefore associated with high-velocity bullets more commonly found in a military setting [6]. Contrary to this, if the patient is stabbed and the object, such as a blade, is left in situ, it can potentially have the protective benefit of providing a tamponading effect, slowing hemorrhage. The physics behind the different offending mechanisms can explain the significantly varied survival rate between gunshots and stab wounds.

The right ventricle is the most common chamber of the heart to be injured, given that it is the most anterior [2]. All penetrating cardiac injuries inherently possess an element of hemorrhage; therefore, they can present along a spectrum of hemodynamic instability. If the pericardial sac is intact, it can play a role in the survival of the patient. A pericardial tamponade will aid in hemorrhage control, slow exsanguination, and allow time for surgical intervention. A retrospective study found that in the setting of penetrating cardiac injury, there is a survival rate of 77% vs. 11% in patients with a cardiac tamponade vs. those without a tamponade [7]. In the presence of a tamponade, Beck’s triad (muffled heart sounds, elevated jugular venous pressures, and hypotension) is pathognomonic. However, recent studies have found that the sensitivity of finding all three signs is 0%, while only one finding was 50% [8]. In our case, upon presentation to the emergency department, the patient was not found to have any evidence of tamponade clinically or radiologically. This is most likely because the injury occurred just before the presentation, and the tamponade did not have time to develop at that point in time. Pericardial tamponade should, therefore, be kept in mind in the setting of a clinically stable patient with a potential cardiac injury that subsequently becomes hemodynamically unstable.

Diagnostic investigations should be tailored to the status of the patient and kept minimalistic, with sonography and X-rays being the most reasonable and efficient investigation modalities. During the repair of the atrial laceration, a running horizontal mattress technique was utilized to minimize the tearing of the thin-walled atria. In a more well-stocked facility, cardiac stabilizers or a Satinsky clamp could have been used to stabilize the operating field of the heart. However, these instruments were not readily available, and the repair had to be performed while the heart was actively beating. Adenosine was not readily available either, which would have been useful while performing cardiorraphy [9]. This lack of resources made a high-risk procedure even more difficult. This is not inclusive of the fact that, if needed, extracorporeal membrane oxygenation and resuscitative endovascular balloon occlusion of the aorta are not available at the facility.

Traumatic cardiac injuries remain a significant source of mortality in developing countries, usually due to their association with violent criminal activities [10]. A previous case report has highlighted that management of patients with penetrating cardiac injuries is indeed possible in the setting of limited resources, although this specific case was complicated by mediastinitis [11]. It is, therefore, no surprise that studies on penetrating cardiac injuries in the developing world focus on charting the clinical outcomes [12]. This can especially be seen with the proposal and validation of the Bogota system, which has been tailored for use in developing countries [13]. This classification system was developed to better streamline the management of penetrating cardiac injuries in low-resource settings that may not have prior well-defined trauma systems. By utilizing basic vital signs and emergency room resources, appropriate staging and treatment can be rapidly performed, increasing patient survival. This is exceptionally useful in a developing country that may witness higher crime rates and, therefore, a higher volume of penetrating cardiac injuries as compared to a more developed country.

It is, therefore, ideal that surgeons with experience in the management of these injuries be available for these procedures. However, in developing countries, there remains an overall lack of specialists [14,15]. This is especially true in the Caribbean, where many general surgeons still manage trauma, cardiothoracic, hepatobiliary, or colorectal patients. This highlights the "all-rounded" nature of general surgeons in the Caribbean. A variety of factors, such as brain drain and a lack of funding and demand, stunt the growth of sub-specialist fields in the region. Even though the "general surgeon" might be lost in the modern age of sub-specialization in developed countries, they still play a major role in the healthcare setting in the Caribbean.

This article is limited by the fact that it is a case report with a patient population of one. While previous studies have looked at the outcomes of patients with penetrating cardiac injuries, they are limited in number. Significantly more research from Caribbean healthcare institutes is needed. The patient did indeed have a positive outcome; however, this case was unique in that there were no significant complications post-operatively. It should also not be misunderstood that while we demonstrate the role and critical importance of general surgeons within the Caribbean, we cannot grow our healthcare systems without an increase in the number of sub-specialists, such as cardiothoracic surgeons. This remains a problem with no clear immediate solution.

## Conclusions

Within the evolving world of surgery, penetrating cardiac injury and its management remain one of the most morbid pathologies to encounter. With a correlation to violent crime, often surgeons in developing countries encounter these injuries without specialist support and equipment. It is, therefore, important to build a solid volume of successful cases that can be used to educate surgeons who may find themselves in similar situations to ours. This case shows that penetrating cardiac injury can be successfully managed operatively by general surgeons, even at low-resource centers in a developing country, through prompt surgical intervention.
